# A score system for quality evaluation of RNA sequence tags: an improvement for gene expression profiling

**DOI:** 10.1186/1471-2105-10-170

**Published:** 2009-06-06

**Authors:** Daniel G Pinheiro, Pedro AF Galante, Sandro J de Souza, Marco A Zago, Wilson A Silva

**Affiliations:** 1 Departamento de Genética, Faculdade de Medicina de Ribeirão Preto, Universidade de São Paulo, Ribeirão Preto, SP, Brazil; 2 Departamento de Clínica Médica, Faculdade de Medicina de Ribeirão Preto, Universidade de São Paulo, Ribeirão Preto, SP, Brazil; 3 Centro de Terapia Celular, Hemocentro de Ribeirão Preto, Ribeirão Preto, SP, Brazil; 4 Ludwig Institute for Cancer Research, São Paulo, SP, Brazil; 5 Decision Systems Group, Brigham and Women's Hospital, Harvard Medical School, Boston, MA, USA

## Abstract

**Background:**

High-throughput molecular approaches for gene expression profiling, such as Serial Analysis of Gene Expression (SAGE), Massively Parallel Signature Sequencing (MPSS) or Sequencing-by-Synthesis (SBS) represent powerful techniques that provide global transcription profiles of different cell types through sequencing of short fragments of transcripts, denominated sequence tags. These techniques have improved our understanding about the relationships between these expression profiles and cellular phenotypes. Despite this, more reliable datasets are still necessary. In this work, we present a web-based tool named S3T: Score System for Sequence Tags, to index sequenced tags in accordance with their reliability. This is made through a series of evaluations based on a defined rule set. S3T allows the identification/selection of tags, considered more reliable for further gene expression analysis.

**Results:**

This methodology was applied to a public SAGE dataset. In order to compare data before and after filtering, a hierarchical clustering analysis was performed in samples from the same type of tissue, in distinct biological conditions, using these two datasets. Our results provide evidences suggesting that it is possible to find more congruous clusters after using S3T scoring system.

**Conclusion:**

These results substantiate the proposed application to generate more reliable data. This is a significant contribution for determination of global gene expression profiles. The library analysis with S3T is freely available at . S3T source code and datasets can also be downloaded from the aforementioned website.

## Background

One of the major challenges in the post-genomic era is the understanding of the genetic basis of gene expression regulation. This involves the deciphering of molecular mechanisms that governs the establishment and maintenance of cellular phenotypes, which has led to a new area of research named "functional genomics" [1] referring to a comprehensive analysis at the protein (proteome) and RNA levels (transcriptome) of any cellular phenotype associated with the expression of whole sets of genes. This is characterized by high throughput or large-scale experimental methodologies combined with statistical and computational approaches.

The correlation between mRNA and protein expression is typically not strong, as previously reported [2]. On the other hand, transcription is one of the most important steps in gene regulation, and information about transcript levels is important to estimate gene activity and to characterize a molecular signature for a cellular phenotype. In this context, new methods have been developed for transcriptome analysis and gene expression profiling [3].

Serial Analysis of Gene Expression (SAGE) [4] is one of the widest used techniques for this purpose. SAGE method allows a quantitative and parallelized analysis of a large number of gene transcripts in any particular cDNA library derived from cells or tissues [5], without prior knowledge of the genes. The SAGE technique is based on the isolation of short sequence tags that are extracted from defined positions of the transcript (3'-most anchoring enzyme restriction site; *NlaIII *is the most commonly used enzyme for this purpose). These tags, in theory, are strongly associated with individual genes [6]. The tags are concatenated into long DNA molecules, which subsequently are identified by conventional DNA sequencing. Sequence tags are extracted from raw sequence concatamers using a parsing program that tabulates the occurrence of each tag and creates an abundance level report file.

Although being advantageous in many aspects [3], SAGE protocol can be also subject to sequence errors, mainly introduced by the polymerase chain reaction (PCR) and sequencing steps. The occurrence of these errors produces sequence-based artifacts, introducing noise into the sampled transcriptome profile. Massively Parallel Signature Sequencing (MPSS) [7] is another sequencing-based method that, similarly to SAGE, provides quantitative gene expression data. This technique can generate millions of short signature sequences, providing a better coverage of the transcriptome in a single cell population. It has a routine sensitivity on a low-molecular level of mRNA per cell.

Another recent technology with high sensitivity is DeepSAGE [8], which is similar to the initial steps of LongSAGE [9] in conjunction with an ultra-high throughput DNA sequencing [10].

In recent years, significant efforts have been directed towards the development of new and reliable DNA sequencing technologies [11]. All three commercially available technologies today are characterized by the production of millions of reads in a single experiment [12,13], reducing the cost of sequencing.

These technologies are available for quantitative measurement of gene expression and, as in SAGE, individual mRNAs can be identified through short tag sequences, immediately adjacent to the 3'-most anchoring enzyme restriction site. The throughput reached by these new sequencing technologies allows also a shotgun strategy for the transcriptome.

Although these represent technological advances, there are still many factors that potentially contribute to the production of artifact sequence tags, such as errors introduced through reverse transcription, PCR, and others, inherent to the cloning (when necessary) and sequencing steps, leading to spurious molecular signatures. In order to mitigate these negative traits, several studies were made to define and explore the inherent implications, mainly to the SAGE protocol. In addition, these studies disclosed the impacts of the spurious sequences and proposed efficient algorithms for detection and removal of these tags [14-17]. In this work, we propose a new methodology to index the sequence tags according to their reliability, using a score system. We also present the implementation of this methodology in a web-based tool, initially available for human (*Homo sapiens*) and mouse (*Mus musculus*) data. The main applications of this tool are the evaluation of the sequenced library, and the identification and selection of the reliable tags, which can be considered more effective for gene expression profiling, according to the scoring system. To rank the tags, we established some empirical rules merging relevant parameters, one based on absolute tag frequency and the other, on matches in a set of virtual tag databases. There is still another component associated with the previous, based on a concept of neighborhood [15], *i.e*. abundant tags can contaminate those whose sequence is very close. The virtual tags are predictions of a real tag that might be produced by a SAGE (10 bps for short SAGE, 17 bps for long SAGE), MPSS (13 bps for short MPSS, 16 bps for long MPSS) or SBS (16 bps or 17 bps) experiments using different anchoring enzymes (*NlaIII *or *DpnII*). They are extracted from gene transcript sequences from various sources with some relevant attributes [18], considered in each score rule elaboration. The other rule component, based on the absolute frequency, assumes that the abundance and representation in the set of libraries presupposes more reliability. The automated library analysis process involves a series of searches on S3T database to retrieve relevant source information from corresponding virtual tag for the application of a specific classification rule set (Table 1). Another approach to rank tags according to their nature, using a relational database and a set of different transcript sequence sources, was previously described [19]. However it does not consider the possibilities of tags derived from SAGE adapters or cloning vectors, or tags matching mitochondrial or nuclear genomes, or tags that can be technology-related artifacts, such as generated by sequencing errors. The approach of S3T comprises these analyses, using additional virtual tag datasets and still considers the tag frequencies to rank the tags.

**Table 1:** S3T Default Rule Set.

**Score**	**Source**	**Condition**
-4	Linker (*)	

-3	mRNAs internally primed	*f*(*x*) = 1

-2		*f*(*x*) < 5 and (*N*(*x*) ∩ *T*) ≠ Ø

10	FL cDNAs, 3'most, poly(A)	*f*(*x*) ≥ 10

9	FL cDNAs, 3'most, poly(A)	1 ≤ *f*(*x*) < 10

8	FL cDNAs, 3'most	*f*(*x*) ≥ 5

7	FL cDNAs, 3'most	1 ≤ *f*(*x*) < 5

6	Consensus, 3'most, poly(A)	*f*(*x*) ≥ 5

5	Consensus, 3'most, poly(A)	1 ≤ *f*(*x*) < 5

4	Alt. poly(A)/splicing, > 1 est	*f*(*x*) > 1

3	Consensus, 3'most	*f*(*x*) ≥ 1

2	Alt. poly(A)/splicing, 1 est	*f*(*x*) ≥ 1

1	FL cDNAs, internal Tags	

0		*m*(*x*) > *f*(*x*)

-1		*f*(*x*) = 1

-5	Mitochondrion genome	

-7	Nuclear genome	

-6	Vector pZErO-1 (*)	

-8		

Rule set used in S3T classification process of public SAGE data. The *f*(*x*) represents the absolute frequency for tag x and *m*(*x*) the average frequency for tag x, considering all libraries in our gene expression database, the set *N*(*x*) represents the tag x neighbor tags (that differ from it by a single nucleotide), the *T *set represents the highest frequency tags of the library (top 20%). (* – This is applicable only for SAGE data)

There is an online service platform, publicly available at S3T website, for accessing and performing the referred analysis. The source code is also available for a local installation.

The S3T approach was applied to a public SAGE dataset and, in addition to the statistical comparisons; two hierarchical cluster analyses were performed using current methodologies [20,21]. The resulting clusters were compared and used to evaluate S3T effectiveness in the prevention against some biases related to sequence-based artifacts or possible contaminations (*i.e*. genomic DNA, mitochondrial DNA, vectors or linkers).

## Results and discussion

The system formerly described, concerning the classification process, is available at S3T website. The services provided have been mainly used to select relevant information from long lists of tags, which are hardly meaningful. The entire library data can be submitted for immediate scanning. The whole procedure takes about 5 minutes for a library with 80,000 unique tags. From these results, it is possible to compare the proportions of each score for the unique tags and their frequencies. The interface provides the means to investigate the makeup of individual tag lists for each score. These individual lists contain the tags, as well as their associated genes and descriptions.

**Figure 1:**
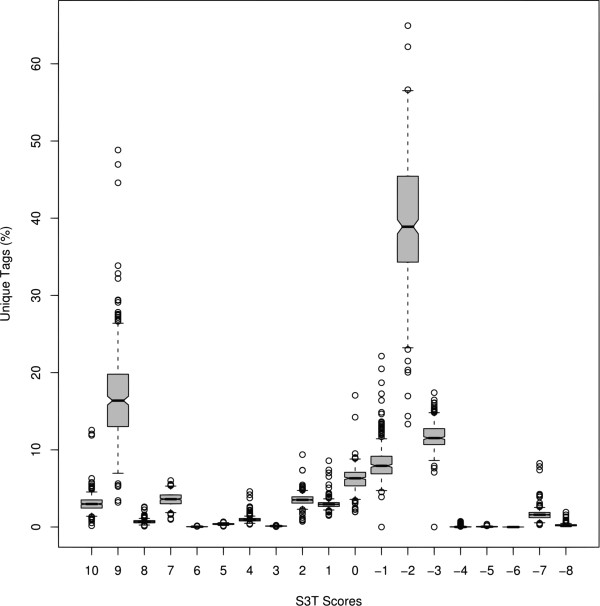
**Distributions of S3T analysis results**. Distributions of unique tag percentages for each score group. This group is formed by tags classified with certain score.

According to the initial analysis using 359 public SAGE libraries, it is possible to observe the large diversity among proportions of tags throughout the established scores (Figure 1). The high percentage of unique tags with -2 score (see Table 1) is evident; they may have arisen from errors in abundant tags whose sequence is very close. This indicates that errors in the tag sequence are the most prevalent. The correlation between the total of sequenced tags in a library and the number of unique tags with -2 score is strong (0.91), *i.e*. the number of unique tags with -2 score increases according to the number of sequenced tags. The correlations between the total of tags in a library and the unique tags in the other scores were obtained and are available at the supplemental section of the S3T website.

**Figure 2:**
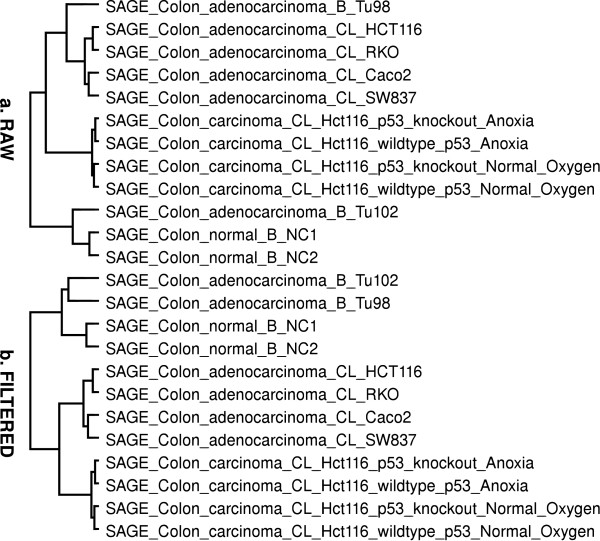
**Cluster analysis of colon SAGE libraries**. Colon SAGE libraries clustered with Pairwise complete-linkage using Euclidean distance of data before (a) and after (b) S3T filtering of tags classified with negative scores.

These results can illustrate the overall quality of tag libraries. Those samples observed with a high percentage of tags classified with negative scores, cannot be considered reliable for further analysis. Box plots were used to visually summarize and compare the groups of data [22] (Figure 1). This diagram contains the distributions of unique tags along the proposed scores. It is crucial to focus particularly on those with positive scores that represent the effective contribution for the biological information, the others, with negative scores, possibly represent contamination or technique errors inherent to the experimental process. However, the tags with negative scores that had matches in the genome sequences, or did not match on anything, could also come from unknown transcripts [23]. But it is also important to emphasize that these unknown transcripts will probably be characterized through the constant updates of the current transcript sequence databases, especially with the use of new high-throughput sequencing technologies, which has high sensitivity to detect rare transcripts. The hierarchical clustering analyses using the selected histological groups (14) of SAGE libraries were also performed. The clusters of samples with known histological information, in a general way, were more concordant with their predefined histological sample classes (see Implementation), after filtering, in some cases (32.65%). In the others, the filtering did not affect the overall quality of cluster set (58.16%) and, in a few cases (9.18%), the clusters after filtering were less concordant with their previously defined classes (Table 2). The brain group is one of them, once there are many heterogeneous samples into subgroups, leading to difficulties to distinguish them among other samples. The F-Measure [24] was used to evaluate the cluster quality. This measure cluster evaluation method combines the precision and recall ideas. The F-Measure values are in the interval [0–1] and larger F-Measure values indicate higher clustering quality. The clusters formed before and after S3T filtering were then compared using the Overall F-measure (Table 2), the expectation was to find clusters more congruous after filtering, properly grouping samples from the same cellular phenotypes together and closer than others (Figure 2).

**Table 2:** Evaluation of Hierarchical Clustering Quality.

**Id**	**Groups (Samples)**	Unique tags * (%)	**Overall F-measure**							
			
			cluster3 [32]/simcluster [21]							
			
			M		A		S		C	
			
				*		*		*		*
1	9(56)	29.21	**0.65**	**0.69**	0.62	0.57	0.55	0.54	0.52	0.52
			
			**0.70**	**0.72**	0.65	0.64	**0.49**	**0.58**		

2	2(7)	29.11	**0.84**	**1.00**	**0.90**	**0.90**	**0.90**	**0.90**	**0.90**	**0.90**
			
			**0.79**	**0.86**	**0.79**	**0.79**	**0.79**	**0.79**		

3	2(24)	30.88	**0.88**	**0.88**	**0.88**	**0.88**	**0.88**	**0.90**	**0.88**	**0.88**
			
			**0.88**	**0.94**	**0.87**	**0.88**	**0.83**	**0.87**		

4	4(12)	33.35	**0.94**	**1.00**	**0.94**	**1.00**	**0.94**	**0.94**	**0.94**	**0.94**
			
			**1.00**	**1.00**	**0.94**	**1.00**	0.90	0.89		

5	2(4)	28.80	**0.83**	**0.83**	**0.83**	**0.83**	**0.83**	**0.83**	**0.83**	**0.83**
			
			**0.83**	**0.83**	**0.83**	**0.83**	**0.83**	**0.83**		

6	2(4)	42.70	**1.00**	**1.00**	**1.00**	**1.00**	**1.00**	**1.00**	**1.00**	**1.00**
			
			**0.83**	**0.83**	**0.83**	**0.83**	**0.83**	**0.83**		

7	14(45)	34.72	**0.72**	**0.74**	0.69	0.64	**0.64**	**0.64**	0.61	0.61
			
			**0.67**	**0.77**	**0.63**	**0.69**	**0.57**	**0.61**		

8	2(5)	27.57	**0.88**	**1.00**	**0.88**	**1.00**	**0.88**	**1.00**	**0.88**	**0.88**
			
			1.00	0.88	1.00	0.88	**0.87**	**0.88**		

9	2(5)	41.48	**0.88**	**0.88**	**0.88**	**0.88**	**0.75**	**0.75**	**0.75**	**0.75**
			
			**0.88**	**0.88**	**0.85**	**0.88**	**0.85**	**0.85**		

10	4(8)	45.00	**0.83**	**0.83**	**0.83**	**0.83**	**0.83**	**0.83**	**0.83**	**0.83**
			
			**0.92**	**0.92**	**0.92**	**0.92**	**0.83**	**0.92**		

11	3(11)	40.70	**0.90**	**0.90**	**0.90**	**0.90**	**0.90**	**0.90**	**0.90**	**0.90**
			
			**0.80**	**0.86**	**0.90**	**0.90**	**0.86**	**0.86**		

12	2(5)	28.30	**0.77**	**0.80**	**0.77**	**0.77**	**0.77**	**0.77**	**0.77**	**0.77**
			
			**0.77**	**0.88**	**0.72**	**0.88**	**0.72**	**0.85**		

13	2(6)	37.71	**0.90**	**1.00**	**0.90**	**0.90**	**0.90**	**0.90**	**0.90**	**0.90**
			
			**0.80**	**0.80**	**0.80**	**0.80**	**0.80**	**0.80**		

14	5(12)	33.63	**0.83**	**0.83**	**0.83**	**0.83**	**0.83**	**0.83**	**0.76**	**0.76**
			
			**0.73**	**0.78**	**0.76**	**0.79**	**0.71**	**0.73**		

Groups of public SAGE libraries used in the hierarchical clustering analyses performed before and after (*) S3T filtering. Four clustering methods were applied: Pairwise complete-linkage (M), Pairwise single-linkage (S), Pairwise centroid-linkage (C), Pairwise average-linkage (A). The histological groups are: brain (1), cartilage (2), cerebellum (3), colon (4), liver (5), lung (6), mammary gland (7), other (8), ovary (9), pancreas (10), prostate (11), retina (12), stomach (13), white blood cells (14). For each group we have the Overall F-measure values for clusters generated with cluster3 and simcluster, respectively, in the first and second lines. The table cell pairs with values in bold represent cases where there was an improvement in the overall quality of clustering, according to F-measure, i.e. the F-measure was greater (21.43% – cluster3; 47.62% – simcluster) or the quality between clusters did not change (69.64% – cluster3; 42.86% – simcluster) after S3T filtering. The remaining table cell pairs, with values not in bold, represent cases where the F-measure was lower after S3T filtering (8.93% – cluster3; 9.52% – simcluster), *i.e*. where the resulting cluster was less concordant with their previously defined classes.

**Figure 3:**
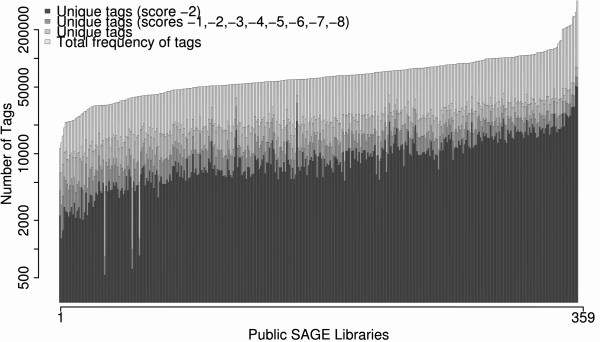
**Contribution of tags with negative scores, especially score -2, in the final library size**. Semi-log graph with unique tags and total frequency of tags for 359 public human SAGE libraries analyzed with S3T, the unique tags and total frequency of tags with negative scores (-1,-2,-3,-4,-5,-6,-7,-8) are also shown in this graph.

In this analysis, applying the proposed set of rules (Table 1), the results (Figure 3) point that the tags with score -2 is the most abundant among those who received negative scores. This was observed for almost all libraries, considering the count of unique tags and the sum of their frequencies (95.26% and 99.16%, respectively). This obviously affects the difference between the raw data and the S3T-filtered data, and indicates which libraries do not have enough tags with good quality; the criterion for this consideration depends on the analysis stringency parameters.

The complete set of results can be found on the supplementary information page at the S3T website, together with other online resources for querying and download.

## Conclusion

SAGE and SAGE-like techniques are useful and efficient methods for quantitative evaluation of gene expression level within a cell population. In addition, the computational approach proposed here enables a noticeable enhancement, in accuracy and efficiency, for these techniques. In this sense, the implementation of this approach provides means for automated analysis and selection of the most reliable tag datasets. In a general way, it was designed to provide researchers with more comprehensive and detailed assessment of a given gene expression profile.

Despite the satisfactory results obtained, one hindrance remains towards tag classification for the process to be deeply related and ingrained to the tag-mapping step. It also depends on the virtual tag database definition and this procedure may lead, in some cases, to tag-to-gene assignments that can be ambiguous, incorrect, or unavailable [25]. This method must be reviewed to improve its accuracy in the presented context. Moreover, the tool allows adjustments to remake the rule sets in order to improve the classification. Additional tests can be made to develop more accurate rules for a precise evaluation of SAGE, MPSS or SBS libraries.

## Implementation

The methodology for quality evaluation of the tag libraries, in a general way, comprises the following fundamental tasks:

1. Collecting experimental data, virtual tag datasets and related information, and store them into the relational database;

2. Setting the score classification rule parameters (Table 1);

3. Carrying out the searches on database and applying the rules, that is a match of the experimental data with one of the tag scoring rules.

4. Quantitating the unique tags and tag frequencies for each score to perform a library quality evaluation.

The classification process is executed in a pipeline mode, applying the rules in a pre-defined order to each and every tag in the library (see Table 1 – from top to bottom). The first attributed score is sustained when the rule is validated; otherwise, the next rule is triggered. A complete set of tools were developed to implement this methodology, which includes the automated process and the website interface. They were developed using Object Oriented Perl programming language and related modules, *e.g. *for database connection (DBI), plot charts (GD::Graph) and interface design (CGI). The S3T system allows multithreading for the classification process, and enables parallel execution on a multiprocessor system. A complete analysis to evaluate the proposed methodology was performed using an experimental SAGE dataset.

### Virtual tag datasets

The sets of virtual tags were obtained from different sources to characterize the experimental data and determine its reliability, based on the relevant characteristics of each sources, as following:

•Cloning vector (*) – sequence of cloning vector plasmid (pZERO-1) DNA;

**• Mitochondrion genome **– sequence of mitochondrial DNA, complete genome (GenBank accession number [GenBank:NC_001807.4] for *Homo sapiens *and [GenBank:NC_005089.1] for *Mus musculus*);

•Linker (*) – Adapter linker sequences used in SAGE library construction and 1-bp variations sequences (edit distance [26] equal to 1);

**• FL cDNAs **– full-length cDNA sequences from MGC [27], RefSeq [28] and "20K set" [18] transcript database;

**• Consensus **– sequences of trEST [29], a consensus sequence database;

**• Nuclear genome **– sequence of nuclear DNA (current build for *Homo sapiens *and *Mus musculus*);

The procedures for tag extraction in these datasets were similar to the SAGE Genie methodology [18]. In addition, we also considered alternative splicing events supported by evidences found through pairwise comparisons of Expressed Sequence Tag (EST) data. The cases where only one individual EST confirmed the event of alternative polyadenylation or splicing were isolated from those with more than one EST confirming the event. The FL cDNAs and Consensus datasets were divided into two subsets based on the presence of poly(A) tail and signal: subsets with both signal and tail and the remaining ones with neither signal nor tail (like [18]). Only sequences with some evidences of their correct orientation were considered. These evidences include a poly(A) tail of at least 5 adenosines or one of the AAUAAA sequence variations (AAUAAA, AUUAAA, AAUUAA, AAUAAU, CAUAAA or AGUAAA) for poly(A) signal, and the results of ESTscan [30] analysis. Possible internally primed polyadenylated cDNA sequences were identified as a way to characterize internal tags resulted from cDNA synthesis priming from a poly(A) stretch other than the poly(A) tail. Alternative polyadenylation events were figured from the alignment of shorter transcript sequences with a poly(A) signal and a poly(A) tail of 5 adenosines that aligned within the longer entry. We also extracted virtual tags from the vector sequence, mitochondrial genomes, nuclear genomes and linkers representing possible contamination with plasmidial DNA, mitochondrial DNA, nuclear DNA and SAGE adapters, respectively.

The main program receives as input parameters, the library file (tag sequences and counts), the platform technology (SAGE, MPSS or SBS), the anchoring enzyme (*NlaIII *or *DpnII*), the tags length and the species acronym (*Hs *or *Mm*). These input parameters define the rule set to be used in the S3T analysis. The online analysis has distinct preconfigured sets of rules. The rule set used in the paper analyses of the public SAGE libraries (Table 1), were defined by the following input parameters: SAGE, *NlaIII*, 10 and *Hs*. The intention of this rule set is to exclude any tag matching SAGE adapters (-4), low frequency tags (*f*(*x*) = 1) matching internally primed dataset (-3), and low frequency tags (*f*(*x*) < 5) similar to the high abundant tags (20%* most frequent*); the purpose is to detect possible technique artifacts; the next eleven score tests (10, 9, 8, 7, 6, 5, 4, 3, 2, 1, 0) are used to identify tags matching known transcript sequences from different sources and reliability; the next score (0) is the last chance to accept tags, if the average tag frequency in the gene expression database is greater than its frequency in the library, which is being evaluated (*m*(*x*) > *f*(*x*)); the next score test is to retain the remained tags with frequency equal to 1, possibly erroneous tags; the subsequent scores (-5, -7, -6) are to test match with mitochondrial, nuclear genome and cloning vector, respectively; the last score (-8) is not a test, it retains the remained tags.

### Experimental SAGE data

The experimental SAGE data used in the evaluation were collected chiefly from SAGE Genie [18], the effort of CGAP SAGE Project to create a comprehensive database of human gene expression [31] and contains several SAGE libraries from normal and tumor tissues or cell-lines. These libraries were constructed by using *NlaIII *as the anchoring enzyme and *BsmFI *as the tagging enzyme as originally described [4]. A set of public SAGE libraries, 319 from CGAP and 40 exclusively from NCBI Gene Expression Omnibus (GEO), distributed among 35 histological groups, were loaded into our local database and submitted as described in this work.

### Data Repositories

There are two in-house MySQL relational databases that are deeply related with the automated process for an entire library evaluation. The main database contains the virtual tags and their source-related information. The virtual tag datasets, described before, with their respective attributes were loaded in there. The tag evaluation process requires a fast and accurate retrieval of the database content to check if the tag attributes satisfy any rule-based classification criteria. The other database, essential for the classification process, contains the experimental data for SAGE, MPSS and SBS, which is used to retrieve the tag frequencies in other libraries. This database was designed to provide a comprehensive and integrative repository for gene expression data and related information. The information in there can be accessed programmatically through a SOAP interface or through a human-readable web interface (see S3T web page). The main database, with virtual tag datasets and the gene expression database, with the experimental data, are regularly updated (every six months).

### Statistical description

All data were divided into two groups, each one respectively corresponding to the tags classified with positive scores and negative scores. The results were described in reports using statistical methods from R statistical package. These reports contain the average of tag frequency, the redundancy (Unique Tags/Frequency of Tags), and the proportions of tags and frequencies in each group. The samples were ordered according to their proportions of tags with negative scores. The differences between unique tags and frequencies of raw and filtered data (after eliminating tags with negative score) were achieved from each group of frequencies (1 to 10 and more than 10). Pearson correlation was used to assess the relationship between these differences.

### Hierarchical clustering analysis

A hierarchical cluster analysis [20] was performed using an open source clustering software [32] on same tissue type samples in distinct biological conditions, typically normal versus tumor, and/or sample preparation (*e.g*. bulk, microdissection, cell line). The analysis used raw datasets for all samples and these same datasets after filtering.

These samples were classified according to their phenotypes (*e.g*. normal, tumor, tumor grade and stage) and/or sample preparation into their histological group, using SAGE Genie information. Single-sample groups were excluded from this analysis.

Clusters were generated using Euclidean distance with four clustering methods: Pairwise complete-linkage, Pairwise single-linkage, Pairwise centroid-linkage and Pairwise average-linkage. Simcluster [21] were also used to generate clusters using these same clustering methods except the Pairwise centroid-linkage, which was not implemented.

### Front-end

An interface for library evaluation using this automatized approach was built using Perl CGI scripts. This interface is contained into the S3T website, it provides useful information to carry out an analysis and presents the results in a user-friendly manner, where it is possible to explore the submitted data. The methodology proposed by SAGE Genie [18] provides the tag-to-gene assignment used in the results page.

The S3T website is available online under Apache web server running on a Linux box (2 × Intel^® ^Xeon^® ^CPU E5410 @ 2.33 GHz).

## Availability and requirements

Project name: S3T – Score System for Sequence Tags

Project home page: 

Operating system(s): UNIX-like Platforms

Programming language: Perl

Other requirements: Perl v5.8.0 or higher, CPAN modules, MySQL 4.1.13 or higher

License: GNU GPL

Any restrictions to use by non-academics: licence needed

## Abbreviations

S3T: Score System for Sequence Tags; SAGE: Serial Analysis of Gene Expression; MPSS: Massively Parallel Signature Sequencing; SBS: Sequencing-by-Synthesis; EST: Expressed Sequence Tag; CGAP: Cancer Genome Anatomy Project; NCBI: National Center for Biotechnology Information; NIH: National Institute of Health; CPAN: Comprehensive Perl Archive Network.

## Authors' contributions

SJS and WASJ conceived the study and the general design of this study. DGP and PAFG did the technical design, the structure development and assembly the virtual tag datasets. DGP conducted the study, carried out the software and website implementation and drafted the manuscript. SJS and PAFG helped to draft the manuscript. WASJ and MAZ supervised and coordinated the study. All authors read and approved the final manuscript.
